# The inhibitory action of plant extracts on the mycelial growth of *Ascosphaera apis,* the causative agent of chalkbrood disease in Honey bee

**DOI:** 10.1016/j.toxrep.2022.03.036

**Published:** 2022-03-29

**Authors:** Patcharin Krutmuang, Julius Rajula, Sarayut Pittarate, Chanchai Chatima, Malee Thungrabeab, Supamit Mekchay, Sengottayan Senthil-Nathan

**Affiliations:** a Department of Entomology and Plant Pathology, Faculty of Agriculture, Chiang Mai University, Thailand; b Innovative Agriculture Research Center, Faculty of Agriculture, Chiang Mai University, Thailand; c Plant Protection, Royal Project Foundation, Muang, Chiang Mai, Thailand; d Rajamangala University of Technology Lanna, Thailand; e Department of Animal and Aquatic Sciences, Faculty of Agriculture, Chiang Mai University, Chiang Mai Thailand; f Division of Biopesticides and Environmental Toxicology, Sri Paramakalyani Centre for Excellence in Environmental Sciences, Manonmaniam Sundaranar University, Alwarkurichi 627 412, Tirunelveli, Tamil Nadu, India

**Keywords:** Honey bee, *Ascosphaera apis*, Oregano, Cinnamon, Plant extracts

## Abstract

*Ascosphaera apis* is a fungal pathogen, which causes chalkbrood disease in bees and is threatening beekeeping worldwide. The demand for organic honey for export has lately heightened hence the biological control is the option. This study aimed at the in vitro evaluation of the potency of plant extracts against chalkbrood disease for the possibility of being employed as a biological control strategy. The results showed that the combination of plant extracts from cinnamon with spearmint, cinnamon with lemongrass, cinnamon with geranium, and cinnamon with palmarosa at a concentration of 25% and 12.5% inhibited mycelial growth of *A. apis* by 100%. This demonstrated the potentiality of combining different plant extracts in controlling this disease. In addition, oregano caused inhibition of up to 100% singly. Conclusively, cinnamon in combination with several extracts has a great potential in curbing this disease while oregano offers an amazing remedy and hence the best formulations should be generated for the beekeeper to utilize.

## Graphical Abstract

**Figure:**
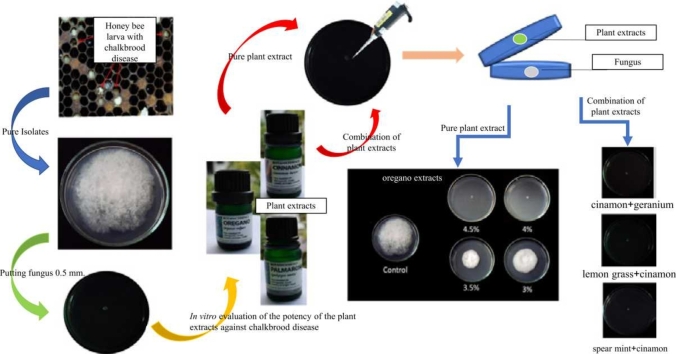

## 1 Introduction

Bees are some of the most important insects in agriculture, firstly, as pollinators and secondly, as producers of the most important source of the sweetest syrup on earth. They emanate from the Order Hymenoptera and Superfamily Apoidea. Honey bees from the genus Apis, however, belong to the sub-family Apoidea. Honey bees are known to pollinate almost 70–80% of plants on earth as the remaining percentage is done by other insects including solitary bees [9]. Stein et al.*,* while carrying out research in Burkina Faso demonstrates that pollination by honey and solitary bees greatly increased the yield capacities and superiority by an average of 62% while on the other hand when not involved, there was an observation of a yield gap of 37% which severely impacts on the food security. The potential bees offer to agriculture, rural employment, and income generation is enormous [21]. The products and by-products of honey bees are useful in a variety of ways. In addition, beekeeping does not require land as the hives are hanged on trees or posts and sometimes placed on rocks. Akratanakul [1] and [7]. Losing bees therefore would mean a paradigm shift in the ecosystem due to their magnanimous role in pollination as demonstrated by [16].

Southeast Asia happens to be the part of the world that is inhabited by the known 10 species of honey bees with Thailand hosting half of these species namely *Apis cerana*, *Apis andreniformis*, *Apis florea, Apis dorsata*, and the introduced *Apis mellifera*. The life of Thais is almost intertwined with traditional honey hunting and beekeeping as reported by Chantawannakul et al. Chantawannakul et al., ($year$) [7]. For several years, the honey produced by the giant and dwarf honey bees has mostly been harvested by hunting while the Asian nest honey bees (*Apis cerana*) are kept in traditional log hives. The introduction of the European honey bee (*Apis mellifora*) came in the company of the modern type of hives known as the box hive [1].

However, bees are presently under great threat from diseases caused by bacteria, fungi, viruses, and predators such as mites which have contributed considerably to colony loss in the recent past [8] and [19]. Honey bee diseases cause huge losses to beekeepers all around the globe. Several fungi have been found to interact with the bees, however, two genera of fungi that are Aspergillus and Ascoesphaera take the lead [20]. That notwithstanding, nosemosis caused by *Nosema apis* has been associated with honey bees for a long time and was reported as early as 1909 by Zander [2]. *Ascosphaera apis* (Massen ex Claussen) Olive and Spiltoir, is a fungal pathogen, which causes a malady in bees known as chalkbrood of bees. Once the larvae of bees which are about 4 days old ingest the spores of the fungi, infection occurs almost immediately. This disease is evidenced by the larvae undergoing invasive mycosis and once dead, they are seen to be covered in a fluffy white mold which later on dry and turn black [20], [3] and [17]. This disease is reported to be more prevalent during spring when the hives are cold and wet giving the fungi a conducive environment to thrive. With the death of larvae in the hive, there could be a proliferation of more spores that would end up infecting most of the bees in the hive which is disastrous. Hedtke et al., in 2011 reports that during spring, as the hives get invaded by *Verroa* mites and *Nosema ceranae*, there is an upsurge of chalkbrood disease [12]. Chalkbrood for a long time has been seen as innocuous hence not given much attention that it deserves. Initially, the nurse in charge of the hive would easily eliminate the diseased larvae hence preventing further infection. However, the chalkbrood disease has been documented to cause a lot of damage and colony loss of up to 37%. This when combined with the other possible infections and predators poses a great danger to the honey bee. It is worth noting that in conducive environments, the infection may get higher than expected [3] and [12].

Due to these disease outbreaks that have threatened beekeeping in Thailand and Southeast Asia, farmers sometimes have been at a loss on what to do in terms of control. This is because the global market has a preference for organic products hence using insecticides would significantly affect marketability. These factors are the premise on which this research was set to come up with a biological alternative for controlling chalkbrood disease hence the in vitro evaluation of plant extracts for their possible application in controlling this disease.

## 2 Material and methods

### 2.1 Isolation of the *Ascosphaera apis*

*Apis mellifera* worker honeybee samples were collected from 1010 Apiaries in three Provinces namely Chiang Mai, Lamphun, and Phrae in Northern Thailand, during the period between July 2013 to June 2014. Three hives were chosen at random in each apiary where about 60 honeybee samples were collected from each hive. After that, they were soaked in 75% ethanol for 10 min, dried, and then stored at –20°C for further experiment. The abdomens of sampled honeybees from each hive were cut and soaked in liquid nitrogen before being ground. Thereafter, distilled water was dropped to be used to check the spores of chalkbrood disease under a stereo microscope 400x. The spore concentration was prepared by using Haemacytometer.

### 2.2 *In vivo* testing of the inhibitory ability of 32 plant extracts vapor on the mycelial growth of fungal pathogen of bees (*Ascosphaera apis*)

**Fig. 1:**
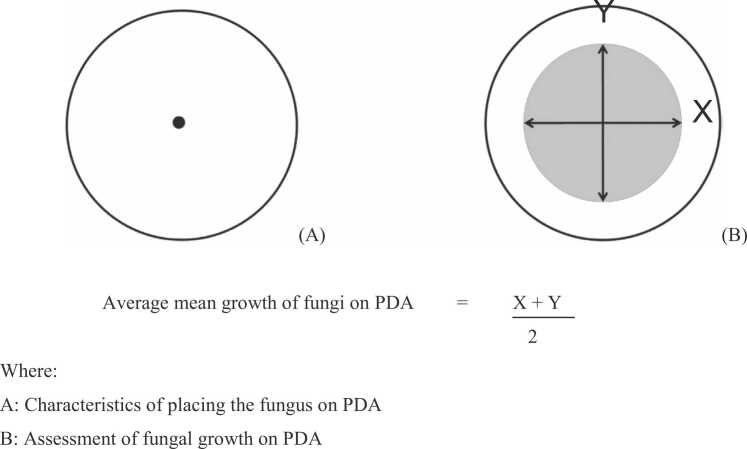
The representation of the effectiveness of plant extract vapors in the inhibition of the mycelial growth of fungus *(Ascosphaera apis)* on PDA. Average mean growth of fungi on $\mathrm{PDA}=\frac{X+Y}{2}$**,** Where: A: Characteristics of placing the fungus on PDA, B: Assessment of fungal growth on PDA.

Thirty-two plant extracts were selected for this study based on their prior knowledge of antimicrobial activities [6]. The efficacy of vapors from 32 plant extracts, namely rosemary *Salvia rosmarinus*, camphor *Cinnamomum camphora*, citronella *Cymbopogon nardus*, peppermint *Mentha x piperita*, elephant grass *Pennisetum purpureum*, orange *Citrus reticulata*, geranium *Pelargonium hirsutum*, eucalyptus *Eucalyptus globulus*, lavender *Lavandula angustifola*, spearmint *Mentha spicata*, cassumunar ginger *Zingiber montanum*, lemongrass *Cymbopogon citratus*, sweet basil *Ocimum basilicum*, coriander *Coriandrum sativum*, fingerroot *Boesenbergia rotunda*, basil *Ocimum basilicum*, ginger *Zingiber oficinale*, bay leaf *Laurus nobilis*, guava leaf *Psidium guajava*, nutmeg *Myristica fragrans*, marjoram *Origanum majorana*, kaffir lime *Citrus hystrix*, palmarosa *Cymbopogon martinii*, tea tree *Melaleuca alternifolia*, turmeric *Curcuma longa*, oregano *Origanum vulgare*, Thai basil *Ocimum basilicum var. thyrsilflora*, phlai *Zingiber cassumunar*, cinnamon *Cinnamomum verum*, black pepper *Piper nigrum*, star anise *Illicium verum* and cajuput *Melaleuca cajuputi* to inhibit mycelial growth of *A. apis* was tested. Fungal culture on potato dextrose agar media (PDA) was prepared by putting the spore at the center of the Petri plate and allowed to grow for 2 days. After that, the plant extracts were prepared by dissolving them in ethyl acetate solvent with 2 concentrations of 25% and 12.5% and then dropping 20 μl of the solution on sterilized filter paper in the lid of Petri plates, which were then allowed some time for the solvent to evaporate. The Petri plate was then turned upside down. The control treatment had a drop of ethyl acetate solvent only. The Petri plates were then incubated at 28 ± 2 °C for 7 days for maximum mycelial growth. The experiment was conducted in a Completely Randomized Design (CRD). Measuring the colony diameter of the fungal on both the X and Y axis was done and then the average percent inhibition of radical growth (PIRG) calculated as follows (Fig. 1) [18].

Formula for calculating the percentage of inhibition of mycelial growth (PIRG)

$$PIRG=\frac{\left(R_{1}-R_{2}\right)x100}{R_{1}}$$

Where R_1_: Radius length of the fungal colony in control.

R_2_: Radius length of the fungal colony in the experiment.

Estimated inhibition values were as follows.

> 75%Very high inhibitory effectiveness.

61 – 75%High inhibitory efficiency.

51 – 60%Moderate inhibition.

≤ 50%Low inhibitory efficiency.

### 2.3 Testing of the efficiency of oregano extract to inhibit the mycelial growth of *Ascosphaera apis*

A drop of *A. apis* culture was made at the center of the Petri plate on potato dextrose agar (PDA). Then, the oregano extracted was dissolved in ethyl acetate solvent of four different concentrations as follows: 4.5%, 4%, 3.5%, and 3%. After that, an aliquot of 20 μl of each of the concentrations was dropped onto a sterile filter paper on Petri plates, and given time for the solvent to evaporate. The Petri plates were then turned upside down with the lid having the filter paper where the oregano extract solution had been dropped. The control however was treated with only ethyl acetate solvent drop on the sterile filter paper. The Petri plate was then incubated at 28 ± 2 °C for 10 days. The experiment followed CRD design. Another evaluation was carried out with oregano at different concentrations but in this case, the extract was dropped directly on 1-day old culture containing *A. apis* on PDA. After that, the extract of oregano was dissolved in ethyl acetate solvents at four concentrations 0.5%, 0.25%, 0.125%, and 0.06%, and then dropped 20 μl of the solution onto the seedling of fungus. The control treatment was a drop of only ethyl acetate then incubated at room temperature for 10 days in CRD design.

### 2.4 The efficiency of vapors of combined plant extracts in inhibiting mycelial growth of *Ascosphaera apis*

The efficiency of combined plant extracts that is: spearmint + lemongrass, spearmint + geranium, spearmint + palmarosa, geranium + lemongrass, geranium + palmarosa, palmarosa +lemongrass, cinnamon + spearmint, cinnamon + lemongrass, cinnamon + geranium and cinnamon + palmarosa in inhibiting the mycelial growth of *A. apis* was also tested*.* One day old culture of *A. apis* on PDA was used. The plant extract was dissolved in ethyl acetate solvents, with two concentrations of 25% and 12.5% and then 20 μl aliquot was dropped onto sterile filter paper on the lid of the Petri plate. This solvent was allowed to vaporize and then the Petri dishes were turned down with the lid where the solution had been dropped. For control, only ethyl acetate solvent was dropped on the sterile filter paper. The Petri plates were incubated at 28 ± 2 °C for 7 days in CRD design.

## 3 Results and discussion

### 3.1 The efficiency of plant extract vapors in inhibition the mycelial growth of *(Ascosphaera apis)* pathogens of bees

**Fig. 2:**
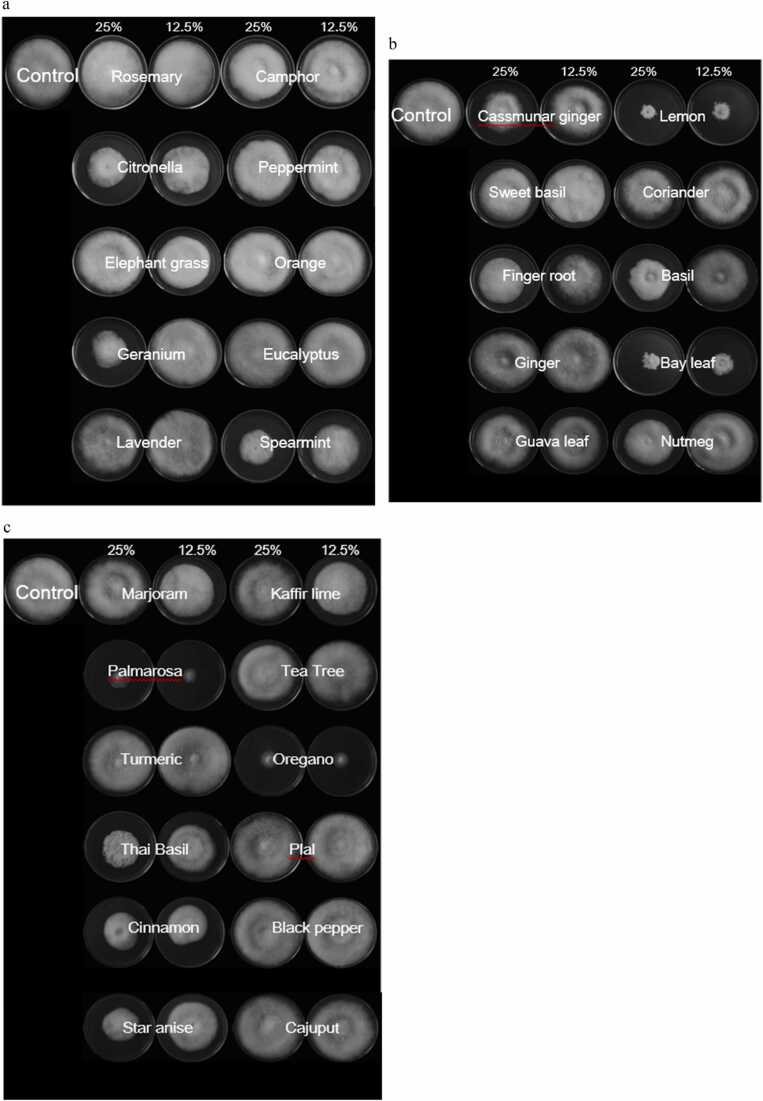
**a** Efficacy of vapors plant extract to inhibition mycelial growth of *Ascosphaera apis* for 7 days, **2 b**Efficacy of vapors plant extract to inhibition mycelial growth of *Ascosphaera apis* for 7 days, **2c** Efficiency of vapors of plant extract to inhibition mycelial growth of *Ascosphaera apis* for 7 days.

The efficiency of vapors from 32 crude plant extracts to inhibit the mycelial growth of *A. apis* was studied at two different concentrations. The results showed that extracts from bay leaf, lemongrass, palmarosa, and oregano at concentrations of 25% and 12.5% were the most effective in inhibiting the mycelial growth of *A. apis* with the percentage range of between 51.11% and 76.48% which was very effective. This result is impressive with the continuous push for organic honey and being cognizant of the threat that synthetic chemicals pose to beekeeping. This result is very impressive when compared to the one done using oregano and thymol on *Aspergillus niger*, *Aspergillus flavus*, *Penicillium* sp., *Fusarium* sp., and *Mucor* sp*.* and the results obtained were way below. The inhibition recorded was below 50% [5]**.** Ficker et al., in 2003 while conducting a study on plant extracts that have antifungal activities against a variety of human fungal pathogens observed the possibility of utilizing these extracts for possible biological control as they do not pose any threat to human which is akin to this study (Table 1 and Figs. 2a–c) [10].

**Table 1:** Efficacy of vapors of 32 crude plant extract in inhibition growth of mycelium of *Ascosphaera apis* on potato dextrose agar for 7 days.

Plant extracts	Concentration (%)	Percent inhibition mycelium^1^
Rosemary	25	14.81 f^2^
	12.5	15.37 f
Camphor	25	22.22 de
	12.5	17.04 de
Citronella	25	54.81 BCE
	12.5	45.56c
Peppermint	25	21.48 de
	12.5	32.22 cd
Elephant grass	25	9.63 f
	12.5	24.63 de
Orange	25	10.93 f
	12.5	13.52 f
Geranium	25	49.26 BCE
	12.5	17.59 de
Eucalyptus	25	7.04 f
	12.5	9.43 f
Lavender	25	15.55 f
	12.5	12.96 f
Spearmint	25	52.78 BCE
	12.5	27.40 de
Cassumunar ginger	25	28.15 d
	12.5	21.48 de
Lemon grass	25	76.48 a
	12.5	61.48 b
Sweet basil	25	28.15 d
	12.5	16.66 de
Coriander	25	28.89 d
	12.5	15.61 e
Fingerroot	25	33.70 cd
	12.5	33.33 cd
Basil	25	33.70 cd
	12.5	33.33 cd
Ginger	25	8.33 f
	12.5	10.93 f
Bay leaf	25	64.45ab
	12.5	51.11 BCE
Guava leaf	25	20.37de
	12.5	17.22de
Nutmeg	25	35.5 cd
	12.5	8.15 f
Marjoram	25	19.63de
	12.5	22.22de
Kaffir lime	25	6.67 f
	12.5	14.26 f
Palmarosa	25	57.41 BCE
	12.5	70.93ab
Tea tree	25	8.89 f
	12.5	10.93 f
Turmeric	25	0.00 g
	12.5	3.33 f
Oregano	25	66.48ab
	12.5	72.04ab
Thai basil	25	37.22 cd
	12.5	20.56de
Phlai	25	0.00 g
	12.5	0.00 g
Cinnamon	25	42.78 cd
	12.5	20.56de
Black pepper	25	0.00 g
	12.5	0.00 g
Star anise	25	38.33 cd
	12.25	21.85de
cajuput	25	0.00 g
	12.25	0.00 g
F-test		***
CV (%)		29.33
LSD 0.05		12.25

*** = Significant at P < 0.001

^1^Average percentage from 3 replications.

^2^The average followed by the same letter in the same column, with lowercase letters analyzed vertically. And showed no significant difference compared to Least Significant Difference at 95%

### 3.2 Testing by drop the oregano extract on the fungus *(A. apis*)

**Fig. 3:**
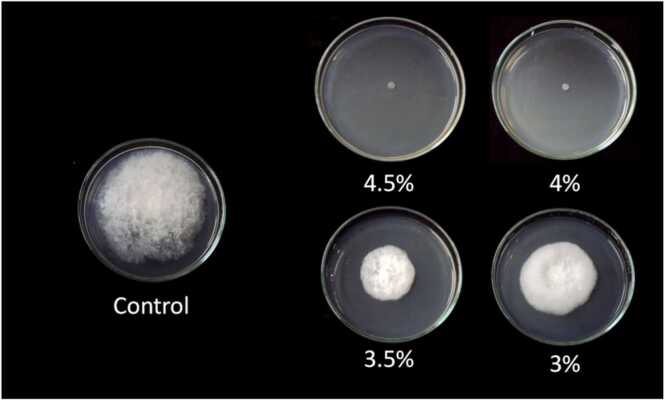
The efficiency of oregano extracts in inhibiting the mycelial growth of *A. apis* for 7 days.

**Fig. 4:**
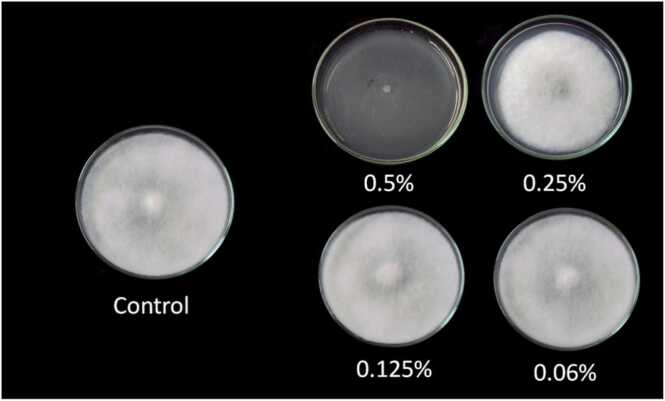
Efficiency of oregano extract in inhibiting the mycelial growth of *Ascosphaera apis* for 7 days).

When the vapors of oregano extract were evaluated, only the higher concentrations of 4.5% and 4% gave 100% inhibition while the concentration of 3.5% and 3% had 47.11% and 31.78% respectively. However, when dropped directly on the fungus, oregano concentration 0.5% caused the highest percentage inhibition of mycelial growth (100%) which was very effective in inhibiting the mycelial growth of *A. apis*. Interestingly, a concentration of 0.25% caused inhibition of up to 9.44% while the concentration of 0.125% and 0.06% did not give significant inhibition to the mycelial growth of fungus (Table 2, Table 3; Fig. 3, Fig. 4). A study carried out in 2012 where oregano was used to test for its inhibitory activities in *Fusarium* and *Penicillium* species caused tremendous inhibitory activities as the number of days increased and the increase in the concentration also contributed. However, it is important to note that the results obtained here are exquisite because of the achievement of the 100% inhibition while in the study performed on *Fusarium* and *Penicillium* species, the highest inhibition activity was 88.85% [14]. In 2018, a study was done in Brazil using nanoemulsions encapsulating essential oil of oregano on several fungal pathogens also gave great support to these findings as the oil from oregano caused inhibition on the fungi that were under investigation [4]. The beauty of oregano is that it is used in the food industry and poses no threat to human health hence it would be very vital in controlling *A. apis* in the beehive. In addition, when gaseous contact of oregano was evaluated in Mexico to determine its effect on the growth of *Aspergillus flavus* at different temperatures and concentrations, it exerted impressive antifungal activities [11].

**Table 2:** Efficiency of oregano extract at various concentrations in inhibiting the mycelial growth of *A. apis* for 7 days).

Plant extract	Percentage of inhibition			
	4.5%	4%	3.5%	3%
Oregano	100.00	100.00	47.11	31.78

**Table 3:** Efficiency of oregano extract in inhibiting the mycelial growth of *Ascosphaera apis* for 7 days.

Plant extract	Percentage of inhibition			
	0.5%	0.25%	0.125%	0.06%
oregano	100.00	9.44	0	0

### 3.3 The efficiency of vapors of combination plant extract in inhibition the mycelial growth of *A. apis*

**Fig. 5:**
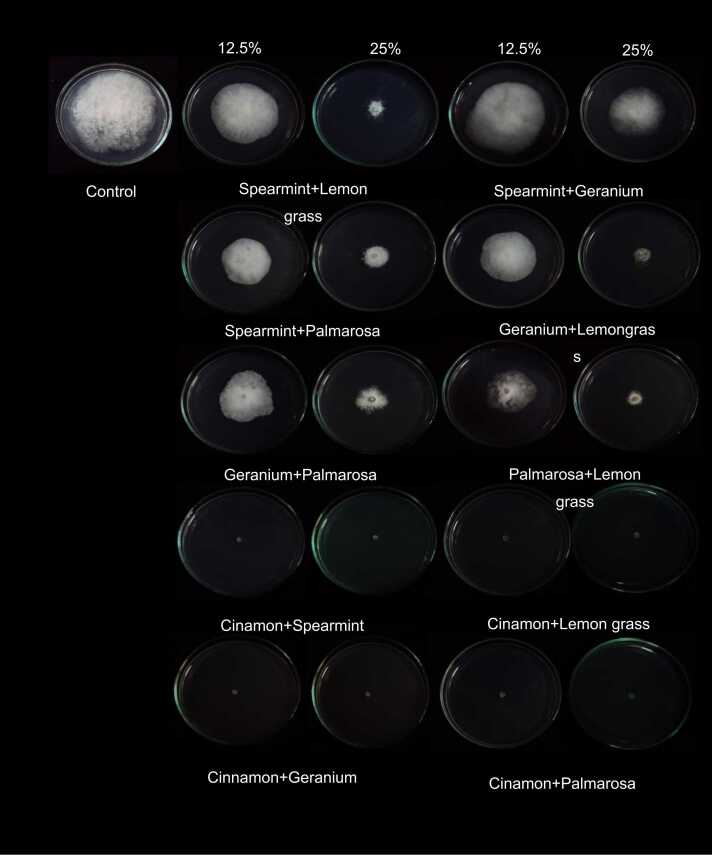
Efficiency of combination plant extract in inhibiting the mycelial growth of *Ascosphaera apis* for 7 days.

When the efficiency of combination plant extracts was evaluated as follows: spearmint with lemongrass, spearmint with geranium, spearmint with palmarosa, geranium with lemongrass, geranium with palmarosa, palmarosa with lemongrass, cinnamon with spearmint, cinnamon with lemongrass, cinnamon with geranium, and cinnamon with palmarosa to inhibit the mycelial growth of *A. apis.* The results show that the combination of plant extracts from cinnamon with spearmint, cinnamon with lemongrass, cinnamon with geranium, and cinnamon with palmarosa at a concentration of 25% and 12.5% can inhibit mycelial growth with the highest percentage of inhibiting mycelial growth of *A. apis* (100%). This was a highly effective inhibition of the mycelial growth of fungi compared to the individual extracts. On the other hand, spearmint with geranium, spearmint with palmarosa, geranium with lemongrass, geranium with palmarosa, and palmarosa with lemongrass was the percentage of inhibition of fungi in the range 20.22–75.11% (Table 4; Fig. 5). When cinnamon was combined with two other plant extracts in 2001, the study obtained outstanding inhibitory effects on bacteria, molds, and yeasts [13]. Also when plant extracts collected from the southern regions of Tanzania were combined and investigated against several fungi to determine their inhibitory potency, the results obtained were of great importance and support the deductions made here [15]. These are indicia of the importance of combining the two or more plant extracts with microbial activities as is an enhancement of their potency and effectiveness.

**Table 4:** Efficiency of vapors of combined plant extract in inhibiting the mycelial growth of *Ascosphaera apis* for 7 days.

Plant extract	Percentage of inhibition	
	12.5%	25%
Spearmint +Lemongrass	31.56 f	72.44 b
Spearmint + Geranium	20.22 g	48.00 df
Spearmint +Palmarosa	52.67 cd	73.33 b
Geranium + Citronella	41.33 e	75.11 b
Geranium + Palmarosa	46.67 de	70.89 b
Geranium + Lemongrass	61.56 c	72.89 b
Cinnamon + Spearmint	100.00 a	100.00 a
Cinnamon + Lemongrass	100.00 a	100.00 a
Cinnamon + Geranium	100.00 a	100.00 a
Cinnamon + Palmarosa	100.00 a	100.00 a
F-test	***	
CV (%)	7.40	
LSD 0.05	4.43

## 4 Conclusions

At the preliminary stage, lemongrass, oregano, bay leaf, and palmarosa gave results of between 51.11% and 76.48%. In the succeeding evaluations, the results reached up to 100% which was amazing. These findings however are primary and require further studies that would elucidate further possibilities of the best way of utilizing these results in coming up with formulations that would be useful to the farmer. The most significant outcome was the potential that lies in the combination of different plant extracts that caused 100% inhibition in most cases. Notably, oregano was able to inhibit mycelial proliferation of up to 100% singly. This is a very impressive finding for oregano is very important as it has been used for a long time in food preservation. Essential oils that are obtained from plants are very important in apiculture with the increased demand for organic honey these hence results are very key in that regard. Conclusively, cinnamon in combination with several extracts has a great potential in curbing this disease, and oregano in its singleness is also an amazing remedy and hence the best formulations can be generated for the beekeeper to be able to use.

## Ethical standards statements

The research did not require any ethical approval and all the co-authors are in agreement with the content of the manuscript.

## Funding

This research received no funding.

## CRediT authorship contribution statement

**Conception and design of study**: Patcharin Krutmuang, Sarayut Pittarate, Chanchai Chantima, Malee Thungrabeab, acquisition of data: Patcharin Krutmuang, Sarayut Pittarate, Chanchai Chantima, Malee Thungrabeab, analysis and/or interpretation of data: Patcharin Krutmuang, Julius Rajula, Sarayut Pittarate, Chanchai Chantima, **Drafting the manuscript**: Patcharin Krutmuang, Julius Rajula, Sarayut Pittarate, revising the manuscript critically for important intellectual content: Patcharin Krutmuang, Julius Rajula, Sarayut Pittarate, **Approval of the version of the manuscript to be published:** Patcharin Krutmuang, Julius Rajula, Sarayut Pittarate, Chanchai Chantima, Malee Thungrabeab.

## Declaration of Competing Interest

The authors declare that they have no known competing financial interests or personal relationships that could have appeared to influence the work reported in this paper.
